# A machine learning model to predict efficacy of neoadjuvant therapy in breast cancer based on dynamic changes in systemic immunity

**DOI:** 10.20892/j.issn.2095-3941.2022.0513

**Published:** 2023-03-24

**Authors:** Yusong Wang, Mozhi Wang, Keda Yu, Shouping Xu, Pengfei Qiu, Zhidong Lyu, Mingke Cui, Qiang Zhang, Yingying Xu

**Affiliations:** 1 Department of Breast Surgery, The First Hospital of China Medical University, Shenyang 110001, China; 2 Department of Breast Surgery, Fudan University Shanghai Cancer Center, Shanghai 200032, China; 3 Department of Breast Surgery, Harbin Medical University Cancer Hospital, Harbin 150081, China; 4 Breast Cancer Center, Shandong Cancer Hospital and Institute, Shandong First Medical University and Shandong Academy of Medical Science, Jinan 250117, China; 5 Breast Center, The Affiliated Hospital of Qingdao University, Qingdao 266003, China; 6 Department of Breast Surgery, Liaoning Cancer Hospital and Institute, Shenyang 110801, China

**Keywords:** Breast cancer, neoadjuvant therapy, peripheral blood lymphocytes, machine learning, prediction model

## Abstract

**Objective:**

Neoadjuvant therapy (NAT) has been widely implemented as an essential treatment to improve therapeutic efficacy in patients with locally-advanced cancer to reduce tumor burden and prolong survival, particularly for human epidermal growth receptor 2-positive and triple-negative breast cancer. The role of peripheral immune components in predicting therapeutic responses has received limited attention. Herein we determined the relationship between dynamic changes in peripheral immune indices and therapeutic responses during NAT administration.

**Methods:**

Peripheral immune index data were collected from 134 patients before and after NAT. Logistic regression and machine learning algorithms were applied to the feature selection and model construction processes, respectively.

**Results:**

Peripheral immune status with a greater number of CD3^+^ T cells before and after NAT, and a greater number of CD8^+^ T cells, fewer CD4^+^ T cells, and fewer NK cells after NAT was significantly related to a pathological complete response (*P* < 0.05). The post-NAT NK cell-to-pre-NAT NK cell ratio was negatively correlated with the response to NAT (HR = 0.13, *P* = 0.008). Based on the results of logistic regression, 14 reliable features (*P* < 0.05) were selected to construct the machine learning model. The random forest model exhibited the best power to predict efficacy of NAT among 10 machine learning model approaches (AUC = 0.733).

**Conclusions:**

Statistically significant relationships between several specific immune indices and the efficacy of NAT were revealed. A random forest model based on dynamic changes in peripheral immune indices showed robust performance in predicting NAT efficacy.

## Introduction

Breast cancer (BC) is the most common malignancy in women and has become an important global public health issue. The diagnosis and treatment of BC have become major clinical goals in recent decades^1^; however, advances in immunotherapy for BC have stalled despite the promising results of immune checkpoint inhibitors in melanoma, non-small cell lung cancer, and bladder carcinoma^2–4^. Although the mutation load and immunogenicity of BC cells are low, several clinical trials of immunotherapy for BC have achieved satisfactory results^5,6^, suggesting that BC is not an isolated zone of immunotherapy. A comprehensive understanding of the changes in immune status during BC progression and treatment is lacking. Accordingly, solid evidence for the effective application of immunotherapy in BC remains insufficient.

Neoadjuvant therapy (NAT) is an important part of the comprehensive treatment for BC. NAT downgrade the stage in patients with locally-advanced cancer and renders inoperable tumors operable. The curative effect of NAT is more intuitive than traditional therapies, thus physicians can adjust the treatment strategy in the early stage and make adjuvant treatment decisions based on the efficacy of NAT. Additionally, NAT provides time for genetic testing of patients and is an active platform for drug research and development. Therefore, evaluation of the efficacy of NAT should not be overlooked. A promising immunotherapy for patients with BC is based on the cytotoxic effects of chemotherapy. Specifically, damaged tumor cells release neoantigens, which increase immunogenicity and transform “cold” tumors with a poor response to immunotherapy into “hot” tumors^6^. Indicators related to the outcomes of neoadjuvant chemotherapy (NAC) have been identified. An analysis combining ECOG 2197 and ECOG 1199 confirmed that a higher number of stromal tumor-infiltrating lymphocytes (TILs) yields better disease-free survival (DFS), distant recurrence–free interval (DRFI), and overall survival (OS) in triple-negative breast cancer (TNBC) patients who underwent adjuvant chemotherapy^7^. A pooled analysis of 3771 patients with BC demonstrated that quantifying TILs before NAT predicts therapeutic efficacy and is related to patient survival, with the exception of patients with luminal HER2-BC^8^; however, the simple single status ‘before NAT’ does not precisely describe patient immune characteristics. The effects of immune status variation on the efficacy of NAT caused by chemotherapy and targeted-therapy agents could provide clues for immunotherapy, but have not been established.

In a previous study we evaluated the prognostic effect of dynamic changes in immune indices by dividing the immune indicator value after NAT by the immune indicator value before NAT. The following post-NAT/pre-NAT immune indicator values were related to the long-term prognosis of patients: CD4^+^:CD8^+^ T cell ratio; CD3^+^CD8^+^ T cell percentage; CD16^+^CD56^+^ natural killer (NK) cell absolute value (Abs); CD3^+^CD4^+^ helper T cell percentage; and summation of T, B, and NK cell percentages [lymphosum (T+B+NK)]. We then used the support vector machine (SVM) to train the prediction model^9^. In this study we focused on the efficacy of NAT and optimized the prediction model using machine learning (ML).

This study retrospectively enrolled 134 women with HER2+ BC and TNBC and recorded immune function indices before and after NAT. We performed univariate and multivariate logistic regression (LR) to select independent indicators. Ten ML methods were used to construct and optimize the final model that described the relationship between these indicators and NAT efficacy.

## Materials and methods

### Study design and setting

We conducted a retrospective study in the Department of Breast Surgery (Cancer Hospital of China Medical University, Shenyang, China) involving 134 patients diagnosed with HER2+ BC and TNBC who had received NAT between 2014 and 2021. The immune indices of patients before and after NAT were collected. This study was approved by the Research Ethics Review Committee of the Cancer Hospital of China Medical University [IRB No. (2019) 2019-72-2, AF-SOP-07-1.1-01]. A total of 17 immune indices were collected as follows: lymphosum (T+B+NK); CD4^+^ helper T cell:CD8^+^ T cell ratio (CD4^+^:CD8^+^ T cell ratio); CD16^+^CD56^+^ NK cell percentage (NK cell percentage); CD16^+^CD56^+^ NK cell Abs (NK cell Abs); CD19^+^ B cell percentage (B cell percentage); CD19^+^ B cell Abs (B cell Abs); CD3^+^ T cell percentage; CD3^+^ T cell Abs; CD3^+^CD4^+^ helper T cell percentage (CD4^+^ Th cell percentage); CD3^+^CD4^+^ helper T cell Abs (CD4^+^ Th cell Abs); CD3^+^CD8^+^ T cell percentage (CD8^+^ T cell percentage); CD3^+^CD8^+^ T cell Abs (CD8^+^ T cell Abs); CD45^+^ cell Abs; total events; reagent lot ID; G2; and lymph events. The **Appendix** figures out the abbreviations of the specific terms in the text and the figures. To clarify, the immune indices noted as “B-” were collected before NAT (B, baseline) and the immune indices noted as “T-” were collected after NAT (T, treated). All patients signed an ethical consent form and approval from the Ethics Committee was received. To comprehensively investigate whether the dynamic changes in the immune indices affected the therapeutic outcome, T-/B- immune indices were calculated by dividing the B- by T- immune indices.

**Figure 1:**
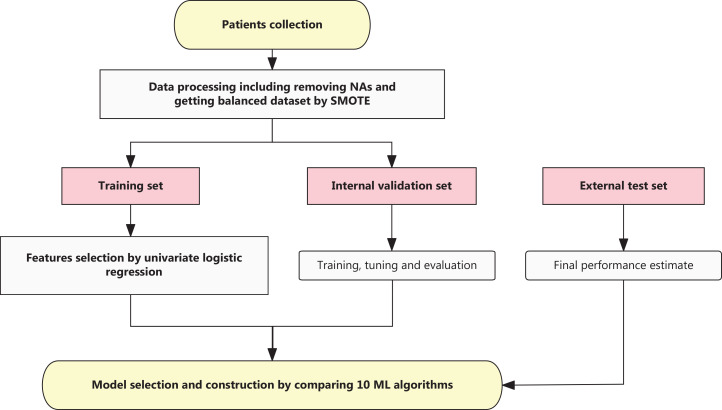
Flowchart describing the study design. Logistic regression (LR) was used in feature selection and machine learning (ML) were performed to construct the model. SMOTE, synthetic minority oversampling technique; NAs, not available values.

The following clinical and pathologic information were collected as follows: therapeutic response; Miller–Payne (MP) grade; Ki67 percentage before NAT; HER2 status; molecular subtype; pathologic N stage at surgery (ypN); clinical primary tumor stage (cT) at diagnosis; and NAT regimen. Histopathologic examinations were performed on tumor biopsy specimens before NAT and the tumor specimens obtained during surgery after NAT. The TNM stages of the patients were defined according to the 8^th^ edition of the AJCC on Cancer TNM staging. Pathological complete response (pCR) was defined as no residual invasive cancer detected by microscopic examination of the excised tumor or lymph nodes after NAT and was used as the prediction target in this study^10^. The flow chart is shown in **Figure 1**.

### Statistical analysis

#### Missing values and balancing outcomes

There were several immune indices with <20% missing values, such as G2 and lymph events, which were imputed using multiple imputation technique based on *mice* R package^11–13^ (version 3.13.0). Due to the imbalance in our data set, the synthetic minority oversampling technique (SMOTE) in the *DMwR* R package^14^ (version 0.4.1) was utilized to generate a new dataset for subsequent analysis. SMOTE is a well-known technique used to handle unbalanced classification problems by artificially generating new examples of minority classes using the nearest neighbors of these cases.

#### Feature selection

To select the essential variables for predicting therapeutic efficacy, univariate LR was used to screen among the 51 immune indices (B, 17; T, 17; and T/B, 17) by setting the cut-off point *P* value at 0.05. Multivariate LR was performed to select the independent predictive peripheral immune indices.

### ML algorithms and model development

ML has been widely applied in biomedicine and clinical medicine for gene discovery, image analysis, and construction of prediction models using heterogeneous data^15^. Based on the ML algorithms supplied by *caret*^16^ (version 6.0.88), 10 methods were utilized to construct and optimize the final prediction model, including random forest (RF), k-nearest neighbors (KNNs), SVMs, classification and regression tree (CART), linear discriminant analysis (LDA), stochastic gradient boosting (GBM), deepboost, multivariate adaptive regression splines (gcvearth), neural network (nnet), and multi-layer perceptron (MLP) with 5-fold cross-validation. Eighty percent of the data was divided into the training set, while the remaining 20% was used as the internal validation set. Box and whisker plots were plotted to evaluate the accuracy and unweighted kappa statistics. The parallel plots visualized high-dimensional data, where each observation was represented by a sequence of its coordinate values drawn according to the coordinate indices. By comparing the accuracy and kappa statistics, we chose the RF estimate as the final model. The number of variables randomly sampled as candidates at each split (*mtry*) was set to one and the number of trees to grow was set to 100. Receiver operating characteristic (ROC) curves and the area under the ROC curves (AUCs) were generated to analyze and calculate the predictive performance of the final model. The importance of the variables was measured based on the *Importance* R function from the *randomForest* R package^17^ (version 4.6.14).

### Software packages

All statistical analyses were performed using R (version: R 4.0.2). The model construction, comparison, and validation were calculated based on *caret* and *pROC*^18^ (version 1.17.0.1) R packages. The SMOTE algorithm was performed in *DWwR* R package. Univariate and multivariate LR were implemented in the *rms*^19^ (version 6.2.0) and *SimDesign* R packages^20^ (version 2.8). The *P*-value cut-off for screening predictors was set at 0.05.

## Results

### Patient characteristics

A total of 101 patients were included in the training and internal validation sets in this study, including 88 (87.13%) patients achieving a non-pathologic complete response (pCR) and 13 (12.87%) achieving a pCR. Data from an additional 33 patients were collected as the external test set (**Table 1**). Among the patients in the training set, 46% were diagnosed with HER2+ BC and 51% were diagnosed with TNBC. Approximately 50% of the HER2+ BC patients in the training set received neoadjuvant anti-HER2 targeted therapy, while all HER2+ patients in the external test set received targeted therapy. With respect to the therapeutic response of the lymph nodes, no pathologic lymph nodes were detected in > 50% of the patients.

**Table 1:** Patient characteristics

	Training set		Test set		Total	
	*n*	Percentage	*n*	Percentage	*n*	Percentage
Treatment response						
Non-pCR	88	87.13%	22	66.67%	110	82.09%
Achieved pCR	13	12.87%	11	33.33%	24	17.91%
Miller-Payne grade						
G1-G3	62	61.39%	13	39.39%	75	55.97%
G4	22	21.78%	8	24.24%	30	22.39%
G5	17	16.83%	12	36.36%	29	21.64%
Ki-67 index (percent)						
≤ 20	17	16.83%	4	12.12%	21	15.67%
> 20	84	83.17%	29	87.88%	113	84.33%
HER2 status						
Positive	46	45.54%	23	69.70%	69	51.49%
Negative	53	52.48%	10	30.30%	63	47.01%
Unknown	2	1.98%	0	0.00%	2	1.49%
Subtype						
TNBC	53	52.48%	10	30.30%	63	47.01%
HR-HER2+	43	42.57%	22	66.67%	65	48.51%
HR-HER2+ (at surgery)	3	2.97%	1	3.03%	4	2.99%
HR-HER2 unknown	2	1.98%	0	0.00%	2	1.49%
ypN						
ypN0	52	51.49%	19	57.58%	71	52.99%
ypN1	21	20.79%	8	24.24%	29	21.64%
ypN2	13	12.87%	2	6.06%	15	11.19%
ypN3	15	14.85%	4	12.12%	19	14.18%
cT						
cT1	7	6.93%	2	6.06%	9	6.72%
cT2	72	71.29%	28	84.85%	100	74.63%
cT3	20	19.80%	2	6.06%	22	16.42%
Unknown	2	1.98%	1	3.03%	3	2.24%
Chemotherapy regimen						
Chemo (TEC/TAC)	77	76.24%	10	30.30%	87	64.93%
H/HP-chemo	24	23.76%	23	69.70%	47	35.07%

HER2, human epidermal growth factor receptor 2; TNBC, triple-negative breast cancer; pCR, pathologic complete response; ypN, pathologic N stage after NAT; H, trastuzumab; P, pertuzumab; T, taxanes; E, epirubicin; A, adriamycin; C, cyclophosphamide; chemo, chemotherapy (including TEC/TAC).

### Selected variables

**Figure 2:**
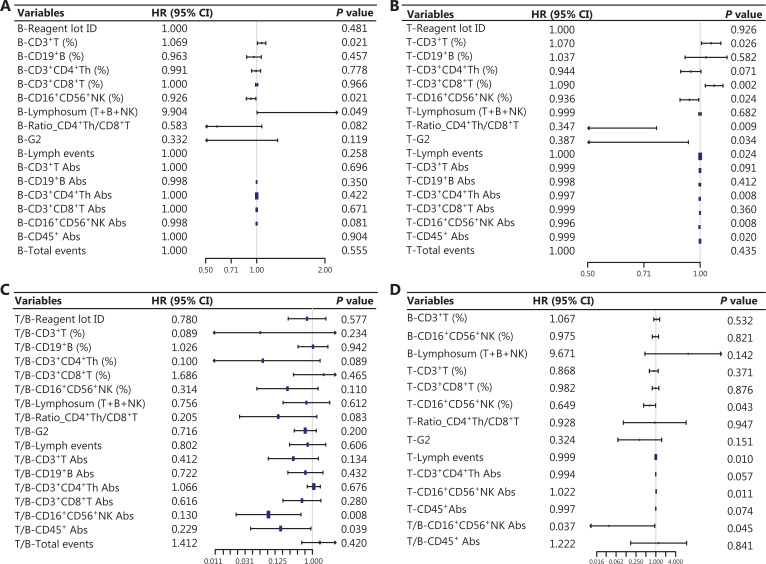
Forest plots showing the results of feature selection: (A) results of univariate LR for the B-immune function indices; (B) results of multivariate LR of the T-immune indices; (C) results of univariate LR for the T/B-immune indices; (D) results of significant multivariate features screened by univariate LR (*P* < 0.05). The forest plots showed the *P* value, hazard ratio (HR), and 95% confidence intervals (CIs) of the immune indices.

The LR results identified several reliable immune predictors (**Figure 2**). After setting 0.05 as the threshold for the *P* value, the B-CD3^+^ T cell percentage (HR = 1.069; *P* = 0.021) and B-lymphosum (T+B+NK) (HR = 9.904; *P* = 0.049) were shown to be positively correlated with pCR. Additionally, **Figure 2B** shows the summary statistics for the significance of the immune indices after NAT. The T-CD3^+^ T cell percentage (HR = 1.07; *P* = 0.026) and T-CD8^+^ T cell percentage (HR = 1.09; *P* = 0.002) were positively correlated with the pCR, whereas a negative correlation was noted between the T-CD4^+^:CD8^+^ T cell ratio (HR = 0.347; *P* = 0.009), T-CD4^+^ Th cell Abs (HR = 0.997; *P* = 0.008), T-NK cell percentage/Abs (HR = 0.936; *P* = 0.024; HR = 0.996, *P* = 0.008), and pCR. The univariate LR of T/B immune indices (**Figure 2C**) showed that T/B-NK cell Abs (HR = 0.13; *P* = 0.008) and T/B-CD45^+^ cell Abs (HR = 0.229; *P* = 0.039) were negatively correlated with pCR, revealing an evident decrease in NK and CD45^+^ cells that could predict a greater probability of achieving a pCR. Taken together, these results show the importance of dynamic changes in peripheral blood lymphocytes, specifically the dynamic depletion of NK cells, adequate reserve of adaptive immune components, and functional activation status of CD8+ T cells (lower T-CD4+:CD8+ T cell ratio) in predicting a pCR. To fully investigate the distinct linear relationship between the immune indices and pCR at different NAT time points, the results of multivariate LR confirmed the solid predictive value of NK cells. T/B-NK cells exhibited a distinct negative relationship with pCR, indicating that persistent consumption of NK cells throughout NAT was essential for achieving a pCR. Finally, 14 variables were selected for the next model construction based on the results of univariate LR. The dynamic landscape of the significantly predictive immune features is shown in **Supplementary Figure S1**.

### Outcomes of the ML models and feature importance

**Figure 3:**
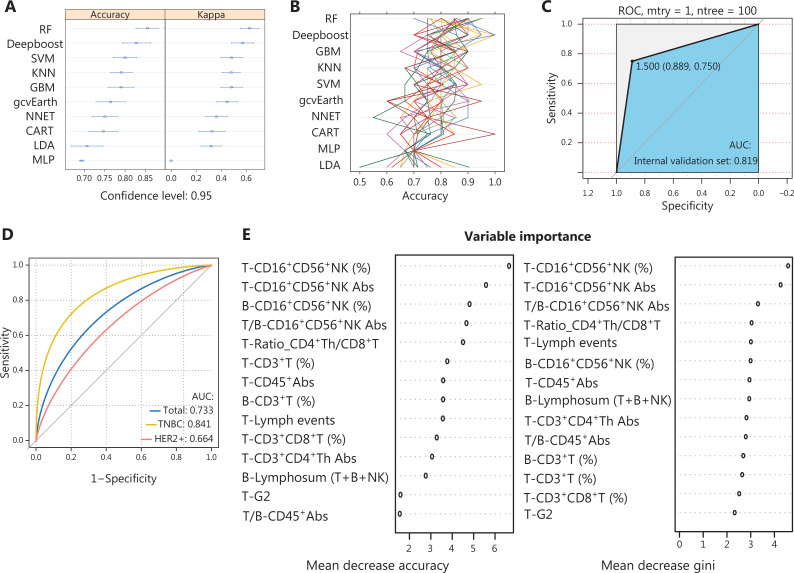
Comparisons among the 10 ML algorithms based on comparing accuracy and kappa: (A) boxplots presenting the accuracies and kappa values of the 5 models; (B) correlation between kappa and accuracy in the 5 models; (C) receiver operating characteristic (ROC) curves when applying the random forest model in predicting the internal validation set; (D) ROC curves when applying the random forest model in predicting the external test sets; (E) importance of features in the random forest models. Mean decreases in accuracy and gini (sorted decreasingly from top-to-bottom) of attributes were as assigned by the random forest. RF, random forest; KNNs, k-nearest neighbors; SVMs, support vector machines; CART, classification and regression tree; LDA, linear discriminant analysis; GBM, stochastic gradient boosting machine; gcvearth, multivariate adaptive regression splines; NNET, neural network; MLP, multi-layer perceptron.

After assessing the predictive performance of 10 ML models (**Figure 3**), the random forest was ultimately chosen because of greater robustness in terms of accuracy and kappa value (**Figure 3A and 3B**), which were validated in the validation set (AUC, 0.819; **Figure 3C**) and the external test set (AUC, 0.73). To explore the predictive performance in different molecular subtypes, we divided the test set into two groups (TNBC and HER2+ BC). The AUC for the TNBC subtype was 0.84 (**Figure 3D**). The dot chart (**Figure 3E**) illustrates the variable importance as measured by the random forest model, visually displaying the specific importance of NK cell percentage/Abs both at surgery and baseline and in the T:B ratio. Notably, NK cells had a considerable role in the random forest model.

## Discussion

Greater than 290,000 BC cases are expected to be diagnosed in the US during 2022, resulting in approximately 43,000 deaths. BC is the most common malignancy among women worldwide^1^. In the post-genomic era NATs continue to have a significant role by providing rapid, direct assessment of treatment effects based on imaging examinations of tumors, as well as research opportunities by comparative studies of tumor biology before, during, and after treatment. The stratification based on tumor-intrinsic subtypes (ER, PR, and HER2) is another attractive strategy that can be incorporated into patient selection for treatment escalation or de-escalation. Based on the heterogeneous biological process in different molecular subtypes, translational research efforts have focused on identifying predictive biomarkers of the therapeutic response, focusing on the primary tumor foci, including aberrant gene expression of tumor cells, TILs, tumor mutational burden, and mismatch repair deficiency. Unfortunately, none of these potential biomarkers have yet reached clinical utility. Therefore, finding biomarkers capable of predicting the response to the diverse innovative treatment approaches, particularly immunotherapy, combined with conventional chemotherapy and pinpointing the change in the immune system during NAT remains extremely difficult tasks. From a mechanistic perspective in cellular biology, most chemotherapeutic agents applied in the clinic eventually affect tumor progression by triggering the pro-apoptotic suicidal machinery of cancer cells. Despite chemotherapeutic potency, escape and resistance are still widely observed in the remaining malignant cells at tolerable doses. In the presence of sufficient leukocyte populations with cytotoxic antitumor immune function, chemotherapy agents can enhance tumor antigen-induced immunogenic cell death. As a less invasive test, examinations of systemic immune components have been long neglected.

In the current study we collected the peripheral blood immune indices from 101 patients with BC before and after NAT, and data from 33 patients were retained as an external test set. Data processing, including k nearest neighbor imputation and SMOTE+, the Wilson’s edited nearest neighborhood (ENN), which is a comprehensive oversampling method proposed by Batista et al.^21^ in 2004, was used to remove not available values (NAs) and obtain balanced datasets. The training set was randomly divided into training and internal validation sets. All immune features in the training set were selected using the univariate and multivariate LR. Fourteen indicators significantly related to the pCR were included in the subsequent model construction. In a previous study, only the SVM was used to build the prediction model; however, to offset the bias caused by a single method and improve the accuracy, we performed KNN, RF, CART, and LDA, for a total of nine ML models, in addition to SVM, to select the best description of the relationship between immune status and NAT response. RF was finally used to construct the immune index model.

Using the above LR processes, NK cells marked as CD16^+^CD56^+^ were significantly related to the efficacy of NAT, whether NK cells were measured before NAT, after NAT, or the after and before NAT ratio. According to the results of the stability and ranking of predictors from the RF variable importance measures, the extreme importance of T/B NK cells also confirmed the results of univariate and multivariate LR, as mentioned above. NK cells have a significant role in innate immunity as cytotoxic lymphocytes by directly killing viruses, bacteria, and cancer cells^22,23^. As illustrated in the current study, the higher the percentage of CD3^+^ T cells before NAT and CD8^+^ T cells after NAT, and the lower the number of T-NK and T/B-NK cells detected in the peripheral blood, the better the patients respond to NAT. Systemic immune status with more adaptive immune components is usually positively related to the pCR, which could be explained by the fact that immunogenic cell death enhanced by chemotherapy agents requires adequate T cell reserves. Interestingly, a decrease in innate immune components, such as NK cells during NAT, leads to a better response. Based on a review of the extant literature, short- and long-term depletion of the main peripheral blood monocyte subtypes (B, NK, and CD8^+^ T cells) were observed after chemotherapy or radiotherapy, likely because the cytotoxic drugs kill tumor cells and at the same time affect the proliferation and survival abilities of systemic immune lymphocytes^24^. A previous study, however, identified tumor-infiltrating NK cells as an independent biomarker for pCR^25^. The contradiction suggests that NK cells infiltrated in the local tumor site operated in concert with T cells to target the tumor cells, while systemic NK cells were affected by chemotherapeutic agents and exhibited a depleted phenotype. The greater the NK cell decline during NAT, the greater the cell death induced by cytotoxic drugs, and the better the tumor response to chemotherapy and targeted therapy.

Immunotherapy has gradually become the most high-profile treatment strategy of the 21st century^26^. The success of immunotherapy has been widely reported in lung carcinomas, and head and neck cancers. Regarding the use of immunotherapy in BC, IMpassion031 provided evidence that immunotherapy was beneficial in the early stages of BC, not just the late stages^5^. Owing to the optimized results of KeyNote-522, the US FDA approved the use of pembrolizumab in combination with chemotherapy for NAT in patients with TNBC. Pembrolizumab monotherapy continues to be used as adjuvant therapy after surgery on 26 July 2021^27^. Additionally, PD-1 inhibitors combined with routine neoadjuvant chemotherapy are recommended for TNBC patients according to the Guidelines of the Chinese Society of Clinical Oncology (CSCO). This news undoubtedly brings light to further research on NAC combined with immunotherapy for BC. Moreover, accumulating evidence suggests that a competent immune function guarantees the patient response to adjuvant biologic treatments, such as trastuzumab, raising the possibility that chemotherapy-induced immune dysfunction render patients less responsive to modern targeted therapies. Similarly, host immune function is critical to the various immunotherapies under development and should be considered if combined with cytotoxic chemotherapy. With respect to the potential application of NK cells in the future, immunotherapy targeting NK cells, including chimeric antigen receptor (CAR)-engineered NK (CAR-NK), adoptive NK cell transfer, and bi- and tri-specific killer cell engagers (BiKEs and TriKEs, respectively), are a promising strategy for hematologic malignancies^28^. Studies focusing on the key effects NK cells exert on BC have grown in number exponentially. Keratin-14-positive BC cells have been shown to prompt NK cells to be less cytotoxic and quiescent, leading to decreased metastasis^29^. Yu et al.^30^ reported that microwave ablation of primary BC inhibits metastasis and prolongs survival via the macrophage/IL-15/NK cell axis rather than CD4^+^ or CD8^+^ T cells. Among patients with advanced BC, the metabolic disorder of circular NK cells can be repaired when TGF-β is inhibited^31^. These results indicate that various NK cell products and related therapeutics have broad applications in the future. To the best of our knowledge, this study extends the suggestions for NAT based on peripheral blood monocytes. The immune index model constructed in the current study may provide references for predicting the efficacy of immunotherapy combined with NAC.

This study had certain limitations. First, evaluation of peripheral blood immune function has only been performed in recent years and is not part of routine care. Complete immune testing data before and after NAC are so valuable resources that large-scale validation is still lacking. Therefore, further prospective or large-scale studies are required to confirm these results. Second, the immune indices included in this study may not represent immune activation status within the tumor microenvironment. More primary tumor tissues corresponding to immune indicators in the peripheral blood need to be collected to enrich the knowledge of immune-related predictive biomarkers.

In this work we identified important indices that were significantly associated with the therapeutic response to NAT in BC and constructed an immune model that accurately described the association. Our research provides valuable information for understanding the panoramic profile of immune status in patients with BC who underwent NAT.

## Conclusions

In the present study we analyzed the correlation between immune indices in the peripheral blood and response to NAT in TNBC and HER2+ patients. Adaptive immune components detected in peripheral blood, including the B-CD3^+^ T cells percentage, T-CD3^+^ T cells percentage, and T-CD8^+^ T cells Abs were positively related to a pCR. As innate immune components, T-NK cells are negatively correlated with a pCR and patients with a significant decrease in peripheral blood NK cells during NAT have a better therapeutic response. We constructed an immune model with high accuracy and specificity based on a RF algorithm by comparing 10 ML models. These findings could lay the groundwork for the application of peripheral blood lymphocyte analysis to predict the therapeutic response of NAC or neoadjuvant targeted therapy combined with immunotherapy in the future.

## Appendix

**Table:**

Specific terms	Abbreviations in figures	Abbreviations in manuscript
Summation of T, B, NK cell percentages	Lymphosum (T+B+NK)	Lymphosum (T+B+NK)
CD4^+^ helper T cell:CD8^+^ T cells ratio	Ratio_CD4^+^Th/CD8^+^T	CD4^+^:CD8^+^ T cell ratio
CD16^+^CD56^+^ natural killer cell percentage	CD16^+^CD56^+^NK (%)	NK cell percentage
CD16^+^CD56^+^ NK cell absolute value	CD16^+^CD56^+^NK Abs	NK cell Abs
CD19^+^ B cell percentage	CD19^+^B (%)	B cell percentage
CD19^+^ B cell absolute value	CD19^+^B Abs	B cell Abs
CD3^+^ T cell percentage	CD3^+^T (%)	CD3^+^ T cell percentage
CD3^+^ T cell absolute value	CD3^+^T Abs	CD3^+^ T cell Abs
CD3^+^CD4^+^ helper T cell percentage	CD3^+^CD4^+^Th (%)	CD4^+^ Th cell percentage
CD3^+^CD4^+^ helper T cell absolute value	CD3^+^CD4^+^Th Abs	CD4^+^ Th cell Abs
CD3^+^CD8^+^ T cell percentage	CD3^+^CD8^+^T (%)	CD8^+^ T cell percentage
CD3^+^CD8^+^ T cell absolute value	CD3^+^CD8^+^T Abs	CD8^+^ T cell Abs
CD45^+^ cell absolute value	CD45^+^ Abs	CD45^+^ Abs
Total events	Total events	Total events
Reagent lot ID	Reagent lot ID	Reagent lot ID
G2	G2	G2
Lymph events	Lymph events	Lymph events
