# Supplementary material for: A machine learning model to predict efficacy of neoadjuvant therapy in breast cancer based on dynamic changes in systemic immunity
Source: Cancer Biol Med. 2023 Mar 24;20(3):218–28. doi: 10.20892/j.issn.2095-3941.2022.0513 (PMC10038070; doi:10.20892/j.issn.2095-3941.2022.0513)
Supplement: Supplementary file 1 [file cbm-20-218-s001.pdf]

Supplementary material

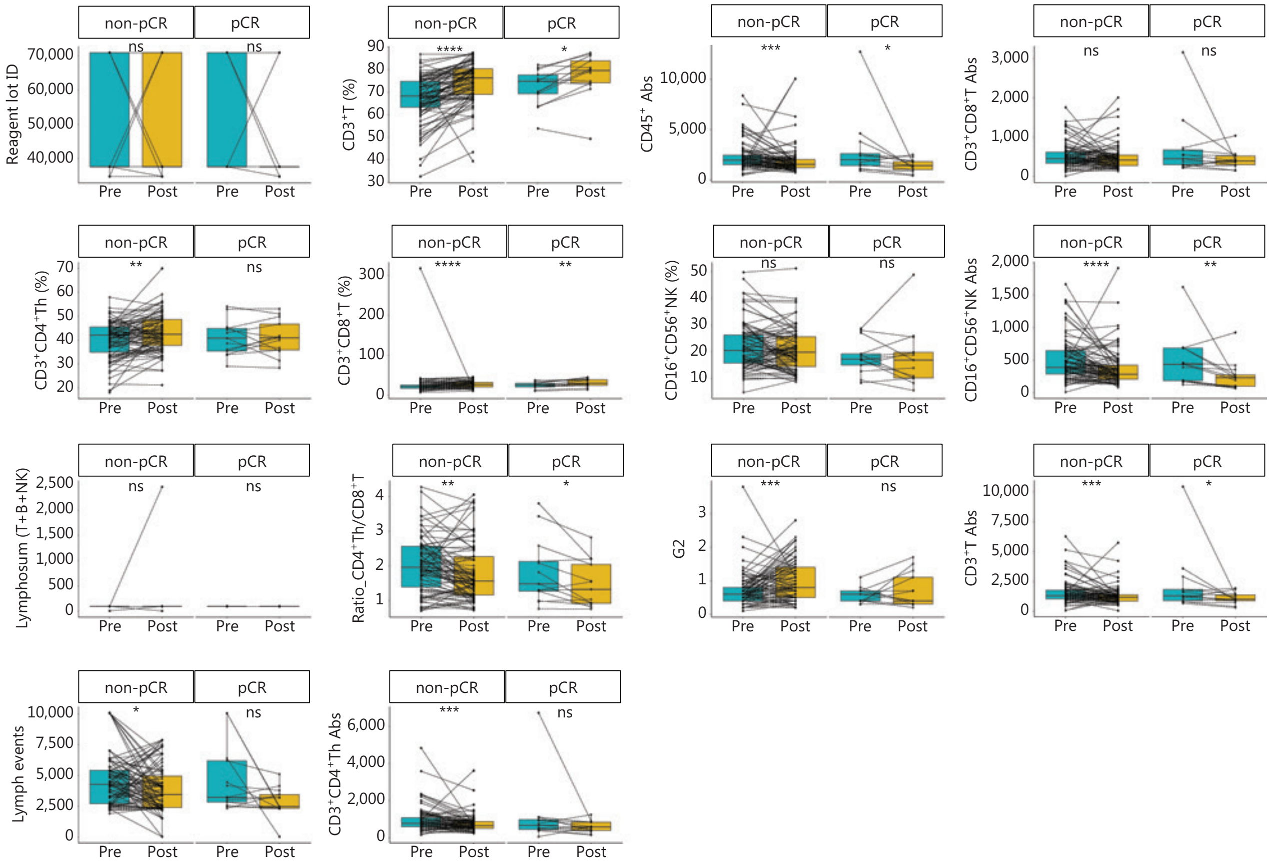

Figure S1 Boxplots of dynamic changes for each immune functional index between the pCR and non-pCR groups. Asterisks (*) represent the level of significance, *P < 0.05; **P < 0.01; ***P < 0.001; ****P < 0.0001; pre, the immune indices before neoadjuvant therapy; post, the immune indices after neoadjuvant therapy.
